# A Novel Method to Study Hip Growth and Development in Children with Cerebral Palsy

**DOI:** 10.3390/children12030367

**Published:** 2025-03-15

**Authors:** Luiz Carlos Almeida da Silva, Yusuke Hori, Burak Kaymaz, Jason J. Howard, Arianna Trionfo, Michael Wade Shrader, Freeman Miller

**Affiliations:** Department of Orthopaedic Surgery, Nemours Children’s Health, Wilmington, DE 19803, USA; la190@duke.edu (L.C.A.d.S.); o21472f@omu.ac.jp (Y.H.); Burak.kaymaz@memorial.com.tr (B.K.); jason.howard@nemours.org (J.J.H.); arianna.trionfo@nemours.org (A.T.); wade.shrader@nemours.org (M.W.S.)

**Keywords:** cerebral palsy, pamidronate, femoral neck isthmus, hip development, hip migration percentage, Gross Motor Function Classification System, proximal femoral guided growth

## Abstract

Background: Knowledge of the relative contributions to different growth areas in the proximal femur and acetabulum is limited due to the complex anatomy and lack of growth markers in children. There is increasing interest in using guided growth to improve hip joint stability and decrease dysplasia in children with neurological disability. Some children with cerebral palsy (CP) are treated with bisphosphonates for bone insufficiency, which leaves a dense growth arrest band in the bone at the time of treatment. The aim of this study was to develop a novel approach to understand the growth and maturation impact on hip development in children with CP using this growth arrest band. Methods: Pelvic radiographs of children with CP Gross Motor Function Classification System (GMFCS) level IV/V treated with bisphosphonate were analyzed. We measured neck–shaft angle (NSA), head–shaft angle (HSA), and migration percentage (MP) based on pamidronate bands (PamMP), NSA based on pamidronate bands (PamNSA), and HSA based on pamidronate bands (PamHSA). These measurements were compared using *t*-test. Results: Seven children (two GMFCS IV and five GMFCS V) were included. The mean age of the radiographic assessment was 11.4 ± 1.3 (range, 8.6–12.5) years, mean MP 22 ± 7% (range, 13–39%), PamMP 33 ± 7% (range, 18–46%), NSA 151 ± 7° (range, 140–161°), PamNSA 153 ± 4° (range, 142–163°), HSA 164 ± 12° (range, 142–175°), and PamHSA 169 ± 8° (range, 154–175°). MP decreased by 10.5% compared with PamMP (*p* < 0.001). NSA compared with PamNSA (*p* = 0.117) and HSA compared with PamHSA (*p* = 0.325) were not statistically different. Conclusions: This novel assessment method demonstrates that ossification of the lateral acetabulum and femoral head in children with CP GMFCS IV/V from age 8 to 12 years undergoes a mean decrease of 10% MP. A decrease of 10% MP after proximal femoral-guided growth has been reported as a positive outcome. However, based on the current measurements, this may be due to normal development. HSA and NSA remained unchanged.

## 1. Introduction

Hip displacement represents a frequent medical problem in children with cerebral palsy (CP) [1,2] and is linearly related to increasing Gross Motor Function Classification System (GMFCS) levels. The risk of a dislocated hip in the entire population of children with CP has been estimated to be between 15% and 20% [3], ranging from 70% to 90% for children with CP GMFCS V [4]. Treating hip displacement in children with CP by proximal femoral-guided growth is becoming a more commonly reported procedure [5,6,7,8]. However, it is currently unclear how much acetabular and proximal femoral growth influences acetabular coverage of the femoral head. Understanding the impact of guided growth on treating hip displacement requires knowledge of the natural development of growth patterns of the proximal femur and acetabulum. While growth of the normal proximal femur during fetal and infant stages is well documented [9,10], the growth patterns during childhood and adolescence are less well studied because it is very hard to accurately measure change. The highest risk of hip displacement is seen in children between the ages of 2 and 5 years who have severe limitations in GMFCS [11,12]. In 2005, Hägglund et al. [3] reported that hips with a mean migration percentage (MP) of 35% may correct to normal without surgery, while those exceeding 42% may not. Additionally, Wordie et al. [13] demonstrated that the critical point for irreversible hip displacement occurs at 46% MP.

Traditionally, proximal femur and acetabulum geometry in children with CP have been evaluated using serial radiographs, which require multiple imaging studies over several years of follow-up, thereby exposing patients to repeated radiation [11]. However, proximal femur growth can be assessed by examining growth disturbance lines, such as those resulting from trauma, infection, chemotherapy, immobilization, and malnutrition [14]. These lines, known as Harris growth arrest lines, serve as biological markers for assessing long bone growth [15]. More recently, sclerotic bands have been observed in children with CP treated with bisphosphonates [16]. These bands correspond to the number of treatment cycles and become visible over time. The distance between these bands and the new bone growth depends on the age of the child and the growth potential of the physis [16,17]. On average, these bands remain visible for 4 years post-treatment and can persist up to 8 years after a pamidronate cycle [17]. Despite extensive research on hip displacement in CP, there remains a significant gap in the literature regarding the quantification of longitudinal hip growth, particularly the relative contributions of the proximal femur and acetabulum during childhood and adolescence, as most existing studies rely on static radiographic assessments rather than dynamic markers capable of tracking growth over time [4,5,7,11,12]. In this context, the use of pamidronate bands as intrinsic biological markers provides a novel approach by offering fixed, time-stamped reference points within the bone. This allows for more accurate assessments of femoral and acetabular growth with a single radiograph compared with traditional serial radiographic methods that depend on external landmarks susceptible to positional variability. To our knowledge, no previous studies have used pamidronate lines as biological markers to evaluate proximal femoral geometry in children with CP using a single radiograph.

This study aimed to assess the growth patterns of the proximal femur and acetabulum during childhood and early adolescence in children with CP GMFCS IV and V using bisphosphonate bands from pelvic radiographs as a biological marker to quantify changes in hip geometry.

## 2. Materials and Methods

**Figure 1:**
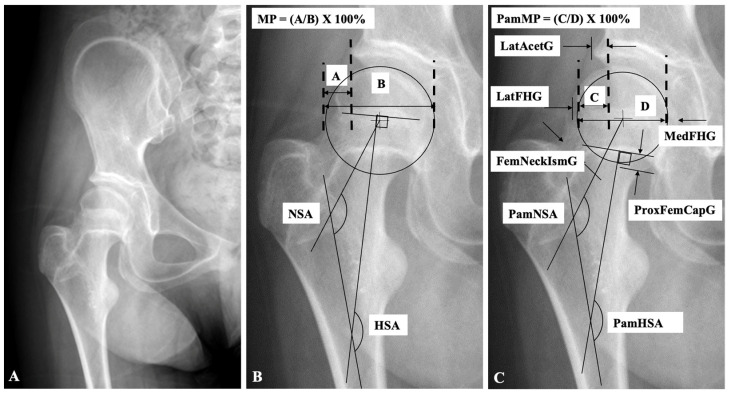
These pelvis and proximal femur radiographs are from a 12.5-year-old girl with cerebral palsy, Gross Motor Function Classification System level V, treated with 8 cycles of pamidronate between ages 8.2 and 11.3 years. The visible dense lines from the pamidronate therapy outline the border of the bone when she started the treatment at age 8.2 years. (**A**) Measurements of her bone shape and hip status at the time of the current radiograph based on measurements of the bone shape. (**B**) Measurements of her bone shape and hip status based on measurements of the dense pamidronate lines. (**C**) FemNeckIsmG, femoral neck isthmus growth; HSA, head–shaft angle; LatAcetG, lateral acetabular growth; LatFHG, lateral femoral head growth; MedFHG, medial femoral head growth; MP, migration percentage based on the border of the bone (A/B × 100%); NSA, neck–shaft angle; PamHSA, head–shaft angle based on pamidronate bands; PamMP, migration percentage based on pamidronate bands (C/D × 100%); PamNSA, neck–shaft angle based on pamidronate bands; ProxFemCapG, proximal femoral capital growth.

We retrospectively reviewed the radiographs of children with CP treated at our hospital from 2009 to 2022. Inclusion criteria were all consecutive children with CP with Gross Motor Function Classification System (GMFCS) level IV or V function treated with pamidronate who had an anteroposterior pelvis radiograph more than 12 months following the final treatment, with visible pamidronate bands in the proximal femur and lateral acetabulum to be used as biological markers (Figure 1). Exclusion criteria were previous proximal femoral osteotomies, pelvic osteotomies, incomplete medical records, and incomplete radiographs. Children who underwent hip surgery prior to the evaluated radiograph were excluded. The Institutional Review Board approved the study protocol (1937542-1) and waived the need for informed consent.

Demographic data and medical history were obtained from electronic medical records. These included sex, date of birth, GMFCS level, age at the time of the radiographic assessment, number of bisphosphonate treatment cycles, age at the first cycle of pamidronate, and age at the last cycle of pamidronate.

Patients with CP with one or more low-energy fractures and low bone mineral density documented by dual-energy X-ray absorptiometry scans of the distal femur were indicated for bisphosphonate treatment using pamidronate. Low bone mineral density was defined by a z-score of less than or equal to −2.0 [18]. Pamidronate medication was administered intravenously at a dose of 0.5 to 1.0 mg/kg per day on 3 consecutive days every 3 months in our outpatient facility, with the 3-day cycle repeated every 3 to 4 months for a minimum of five cycles [19].

Anteroposterior pelvic radiographs were obtained as part of routine hip monitoring at least 12 months following the final pamidronate dose of the treatment plan. The rationale for selecting radiographs taken at least 12 months after the final pamidronate treatment was to allow enough time for bone remodeling and growth to occur beyond the immediate effects of the therapy. This ensures a more accurate assessment of hip development during late childhood and early adolescence. All radiographs were obtained using a standardized anteroposterior pelvic protocol at our institution, with the same radiographic setup, a source-to-image distance of 115 cm, and consistent patient positioning to minimize magnification variability; however, no external radiographic marker was utilized. A standardized position for anteroposterior pelvic radiographs is defined in our facility. The children were lying supine with the pelvis at a horizontal level, ensuring no rotation or tilt. The patellae were faced upward, indicating neutral hip rotation, while the hips were in neutral adduction or abduction to avoid a misrepresentation of the joint segment. A cushion or support was placed under the knees if needed to prevent excessive lumbar lordosis and avoid anterior pelvic tilt to achieve a flat lumbar spine and proper alignment. The following data were recorded from radiographs: hip MP based on the usual bone landmarks (MP), hip migration percentage based on pamidronate bands (PamMP), head–shaft angle based on the usual bone landmarks (HSA), head–shaft angle based on pamidronate bands (PamHSA), neck–shaft angle based on the usual bone landmarks (NSA), neck–shaft angle based on pamidronate bands (PamNSA), lateral femoral head growth (LatFHG), medial femoral head growth (MedFHG), femoral neck isthmus growth (FemNeckIsmG), lateral acetabular growth (LatAcetG), and proximal femoral capital growth (ProxFemCapG) defined as the longitudinal growth of the femoral capital epiphysis (Figure 1).

Frequency distribution was utilized to summarize categorical variables. Mean, standard deviation, and range were used to summarize continuous variables. An independent samples *t*-test adjusted by Bonferroni correction was used to analyze age during the assessment of the radiograph, MP, PamMP, HSA, PamHSA, NSA, PamNSA, LatFHG, MedFHG, FemNeckIsmG, LatAcetG, and ProxFemCapG, with a *p* value < 0.05 considered statistically significant. Pearson coefficients were used to assess the bivariate correlation between PamMP and LatFHG. Correlation strength was interpreted as very weak (0.00–0.19), weak (0.20–0.39), moderate (0.40–0.59), strong (0.60–0.79), and very strong (0.80–1.0) [20]. The intra- and inter-observer reliabilities for the two pediatric orthopedic surgeons involved directly in the measurements are expressed in terms of the intra-class correlation coefficient.

## 3. Results

**Figure 2:**
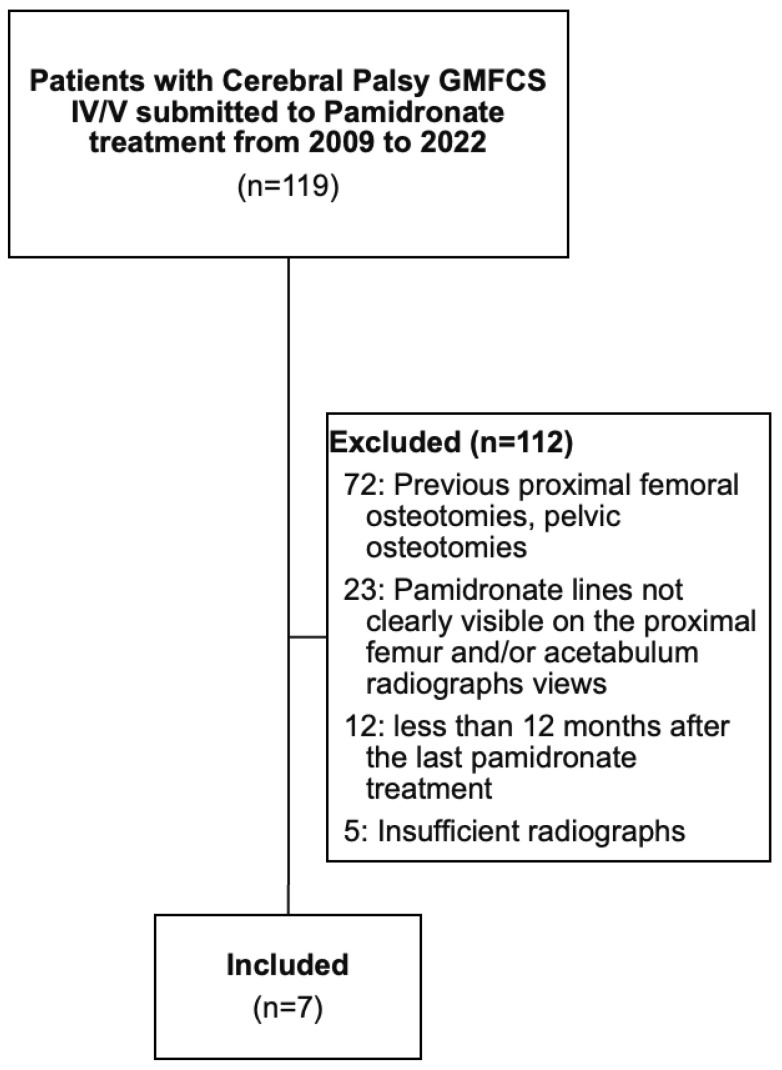
Flowchart of patient selection. GMFCS, Gross Motor Function Classification System.

Seven children met the inclusion criteria (four girls and three boys), and the mean age at the time of the radiographic assessment was 11.4 ± 1.3 years (range, 8.6 to 12.5 years). A flowchart of patient selection is provided in Figure 2. Two children were classified as GMFCS IV, and five were classified as GMFCS V (Table 1). When analyzing the age at the time of radiographic assessment associated with the GMFCS level, the mean age of the children classified as GMFCS IV was 10.4 ± 2.5 years (range, 8.6 to 12.2 years), and the age of those classified as GMFCS V was 11.7 ± 0.7 years (range, 10.8 to 12.5 years) (*p* = 0.269). The mean age at the first cycle of pamidronate for the entire series was 8.1 ± 1.3 years (range, 5.6 to 9.2 years), the mean age at the last cycle of pamidronate for the whole of the series was 10.1 ± 1.3 years (range, 7.5 to 11.3 years), and the mean number of pamidronate cycles was 7 ± 2 cycles (range, 5 to 9 cycles) over a mean of 23.8 ± 4.5 months (range, 15.6 to 28.8 months) (Table 1).

**Figure 3:**
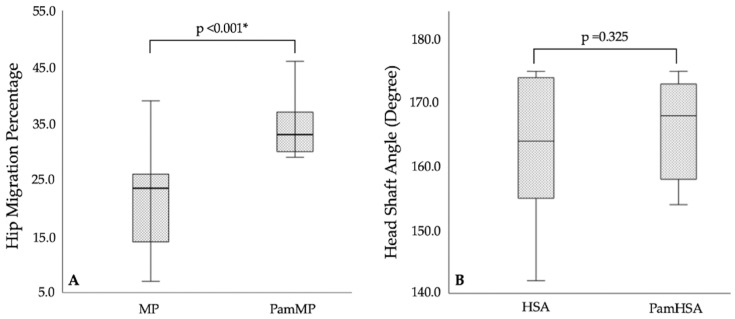
Comparison of two proximal femoral geometry measurements based on the usual bone landmarks and pamidronate bands. (**A**) Hip migration percentage. (**B**) Head–shaft angle. * Indicates a statistically significant *p* value. HSA, head–shaft angle based on the usual bone landmarks; MP, hip migration percentage based on the usual bone landmarks; PamHSA, head–shaft angle based on pamidronate bands. PamMP, hip migration percentage based on pamidronate bands.

The MP decreased by 10.5 compared with PamMP (*p* < 0.001); HSA compared with PamHSA (*p* = 0.325) (Figure 3) and NSA compared with PamNSA (*p* = 0.117) were not statistically different. The LatFHG was higher than the MedFHG (*p* < 0.001), and the ProxFemCapG was higher than the FemNeckIsmG (*p* < 0.001). The LatAcetG was similar to the sum of the LatFHG and MedFHG (*p* = 0.107) (Table 2). Bivariate correlation analyses examined the relationship between MP and LatFHG and demonstrated a very weak relationship and no significant correlation (*r* = 0.043, *p* = 0.875).

Both hips of the same patient were evaluated at the same time (14 hips). The mean MP of the right hips was 18.3 ± 5.7%, and the mean MP of the left hips was 26.3 ± 4.7% (*p* = 0.002). There were no differences between the right hip compared with the left hip for PamMP (*p* = 0.073), HSA (*p* = 0.200), PamHSA (*p* = 0.645), NSA (*p* = 0.810), LatFHG (*p* = 0.162), MedFHG (*p* = 0.240), FemNeckIsmG (*p* = 0.928), LatAcetG (*p* = 0.680), and ProxFemCapG (*p* = 0.611).

The intra-class correlation coefficients for intra-observer reliability ranged from 0.865 to 0.917; inter-observer reliability ranged from 0.801 to 0.886.

## 4. Discussion

Pamidronate bands were utilized as a biological marker due to their clear visibility on radiographs and their non-interference with hip migration. All measurements were made from a single radiograph, thereby removing any errors introduced by position changes that occurred when measuring multiple radiographs over time to assess change. Bisphosphonates, including pamidronate, are primarily designed to inhibit osteoclast-mediated bone resorption, enhance bone density, and reduce fracture rates rather than directly promoting bone growth [19]. Pamidronate does not directly influence muscle tone or the progression of hip displacement in children with CP. Despite extensive research on hip displacement in CP, there remains a significant gap in the literature regarding the quantification of longitudinal hip growth, particularly the relative contributions of the proximal femur and acetabulum during childhood and adolescence, as most existing studies rely on static radiographic assessments rather than dynamic markers capable of tracking growth over time.

The current report is the first to use bisphosphonate bands to evaluate hip growth and development in children with CP GMFCS IV/V. We primarily focused on lateral acetabular and proximal femoral growth by examining pamidronate bands. The main growth areas of the acetabulum are the iliac acetabular, the ischial acetabular center, and the os acetabuli [21,22,23], with the lateral rim of the acetabulum featuring a ring epiphysis that, when damaged, contributes to acetabular dysplasia [24,25,26]. The lateral acetabular ring epiphysis ossifies during adolescence, enhancing hip joint stability in childhood and adolescence [24].

Our findings suggest that early bone development, represented by the pamidronate bands, and the final stages of bone growth, indicated by the usual bone landmarks, were similar for HSA and NSA. This suggests that the proximal femur shape does not change over time. However, differences were observed in the growth of the lateral and medial femoral head, femoral neck isthmus, lateral acetabulum, and proximal femoral capital epiphysis, and in hip migration.

While 30% of longitudinal femoral growth has been typically attributed to the proximal physis and 70% to the distal physis, this ratio is highly age-dependent, with younger children showing a higher proximal to distal growth ratio [22,26]. Critical biomechanical influences include the hip joint reaction force on the proximal femoral growth plate and muscle pull on the trochanteric apophyseal growth plate. These factors significantly impact typical hip joint geometry and growth [22]. The longitudinal and trochanteric growth plate initially appear in infancy as a continuous growth plate [10], radiographically separate in childhood, becoming independent centers of ossification. Some authors have called the area between these growth plates the femoral neck isthmus growth plate [21,22,23], which contributes to lateral neck and longitudinal femoral shaft growth, maintaining pace with the trochanteric and longitudinal growth plates [22]. Our study documents this concurrent and symmetric growth through adolescence (Figure 1). Since no growth in femoral neck width occurs, the medial border of the femoral neck needs to be remodeled similarly to the remodeling of distal femur with growth, which is reportedly impacted by pamidronate treatment [17]. This is also demonstrated by the pamidronate lines showing reduced remodeling in the proximal medial femoral neck between the pamidronate lines and the femoral epiphyseal plate (Figure 1). This effect is hard to measure but appears similar to lack of the cut back seen at the distal femur. Varus and valgus angulation of the femoral neck is controlled by contributions to the lateral growth of the neck by the three growth plates, but the specific impacts of each growth plate are not well defined [23]. Since all three growth plates measured in this study grew relatively symmetrically, there was no change in femoral neck angulation.

This report demonstrates that varying growth and ossification around the hip alter femoral head coverage during maturation (Figure 1). We observed that MP represented by the pamidronate bands was higher compared with MP from the bone outline on the radiograph, showing better femoral head coverage by the acetabulum due to differential ossification of the lateral acetabulum compared with the lateral femoral head. This differential lateral acetabular growth, caused by ossification of the lateral acetabular ring epiphysis relative to the lateral femoral head growth, leads to an approximate 10% MP reduction between the ages of 8 and 12 years. If the hip is stable without progressive femoral head subluxation, femoral head coverage increases during these years. However, these findings are specific to the studied age range and should not be generalized to younger children with CP GMFCS IV/V because of the poor understanding of relative femoral head and acetabular growth ratios at younger ages. The current reported technique of assessing bisphosphonate bands could be applied to understanding the younger hip development; however, we do not have children in the younger ages who meet all our exclusion criteria set out in the current study. A recent systematic review reported an improvement in MP of 8.3% following proximal femoral-guided growth in approximately the same age range [7]. Based on our findings, this change is consistent with skeletal maturation and naturally improved hip coverage and may not be due to the guided growth procedure.

The longitudinal growth of the femoral neck significantly contributes to femoral length and may play a role in the development of coxa valga, commonly seen in children with CP GMFCS IV/V [22]. Data suggest that proximal femoral growth accounts for a more significant percentage of total femoral growth before age 7 years [26], which might explain why femoral valgus primarily develops at a younger age as a response to the mechanical factors [9,22,27]. Our study found no change in HSA or NSA during late childhood/adolescent growth, supporting the notion that the coxa valga stabilizes during adolescence and primarily develops earlier. This aligns with the existing literature linking femoral valgus and hip dislocation in CP [28,29,30].

Our study also found that the lateral femoral head growth was higher than the medial femoral head growth, consistent with findings by Tsukagoshi et al. [31], who demonstrated femoral head flattening in the posteromedial area in dysplastic hips. However, this differential lateral femoral head growth compared with medial femoral head growth is less significant than the increased lateral acetabular ossification, which is the main contributor to decreased MP.

Bisphosphonate bands have been previously used as biological markers to assess longitudinal bone growth and metaphyseal modeling in conditions such as osteogenesis imperfecta, post-chemotherapy bone recovery, and various metabolic bone diseases. They provide insights into growth dynamics and treatment effects over time [16,17]. However, to our knowledge, standardized measurements using bisphosphonate bands to evaluate specific geometric parameters of the hip, such as migration percentage, neck–shaft angle, and head–shaft angle, have yet to be established in the literature. The method employed in this study to assess the proximal femur and acetabular geometry in children with CP represents a novel approach to quantifying hip development in these children. This innovative technique of employing pamidronate bands as intrinsic biological markers offers a distinctive, time-stamped reference for assessing hip growth. It enables single-radiograph evaluations with reduced positional variability, potentially assisting clinicians in differentiating between anticipated developmental increases in migration percentage and the impacts of surgical interventions. This distinction is particularly significant considering the reports of comparable migration percentage improvements following guided growth. Unlike previous studies that have depended on serial radiographs or external landmarks, such as those by Lee et al. [5] and Portinaro et al. [8], this method provides an internal, patient-specific indicator of growth progression. However, we recognized the possibility of observer bias when measuring the positions of pamidronate bands. To address this, standardized imaging protocols and experienced reviewers were utilized to reduce potential discrepancies risk.

Finally, longitudinal femoral growth in the proximal femur is poorly defined [23]; our study suggests that the unified growth plate continues to contribute to femoral growth during childhood and adolescence. Prior studies reported a higher contribution of the proximal growth plate in younger children [26].

Understanding normal acetabular and proximal femoral growth is essential when evaluating the outcomes of proximal femoral-guided growth in managing children with CP and hip dislocation. While we lack corresponding data for children under 8 years old, a 10% MP change between ages 8 and 12 years should be considered normal development if the hip is not actively subluxating and is under an MP of 46%, which was the high MP in our study cohort.

This novel method may influence clinical decision making by providing objective evidence of natural growth-related improvements in hip coverage, which can help distinguish between true treatment effects of guided growth procedures and normal skeletal maturation. Compared with alternative techniques such as serial migration percentage monitoring, three-dimensional imaging, or biomechanical modeling, our approach offers a unique, patient-specific growth reference visible on standard radiographs, although observer bias may still arise from the measurement variability inherent in radiographic analysis.

This study has several limitations, including a small sample size and a limited age range, which restrict the generalizability of the findings. However, patients were selected based on specific inclusion criteria to ensure homogeneity within GMFCS levels IV and V, which severely limited the available study population. Although the sample size of this study is limited to seven children, due to the strict inclusion and exclusion criteria, as well as the rarity of the examined population, this cohort provides valuable insights into hip growth assessment using pamidronate bands. Nevertheless, the small number of participants presents inherent limitations in achieving statistical significance, which should be considered when interpreting the results. As a preliminary investigation, this study aimed to explore the feasibility of using pamidronate bands as biological markers for hip growth. Future research with larger cohorts is necessary to validate these findings and enhance their applicability. Additionally, the retrospective nature of the study may introduce selection bias. Despite this limitation, the use of this novel radiographic assessment technique provides an advantage by minimizing the variability associated with child positioning across multiple radiographs, offering a reliable method to evaluate changes over time.

## 5. Conclusions

This study used a novel assessment based on bisphosphonate bands to study proximal femoral and acetabular growth. Significant increases in growth of the lateral versus medial femoral epiphysis, longitudinal growth of the capital physis versus femoral neck width, and a relative increase in lateral acetabular growth versus lateral femoral head growth led to a 10% MP reduction between ages 8 and 12 years. Substantial persistent lateral tilting of the proximal femoral physis, with associated coxa valga, did not change during this follow-up period. These findings provide insights into the physiological mechanisms underlying changes observed after proximal femoral guided growth procedures for hip displacement in children with CP. Using bisphosphonate bands is a valuable technique for evaluating bone shape changes during growth. Further prospective, multicenter studies are needed to validate these preliminary findings and confirm the applicability of this novel assessment method in larger and more diverse populations.

## Figures and Tables

**Table 1:** Characteristics of the population.

Patient	GMFCS Level	Sex	Number of Pamidronate Cycles	Age at the First Dose of Pamidronate (Years)	Age at the Last Dose of Pamidronate (Years)	Age at Radiographic Evaluation (Years)
1	V	Female	5	9.0	10.3	11.6
2	IV	Male	7	5.6	7.5	8.6
3	V	Female	9	7.2	9.6	11.5
4	V	Male	7	8.1	9.8	10.8
5	V	Female	8	9.0	11.0	12.3
6	IV	Male	9	8.8	11.2	12.2
7	V	Female	8	9.2	11.3	12.5

GMFCS, Gross Motor Function Classification System.

**Table 2:** Radiographic measurements of proximal femoral geometry and bone growing based on the usual bone landmarks and pamidronate bands.

	Mean Value	Minimum Value	Maximum Value	*p* Value
MP (%)	22.7	13.0	39.0	<0.001 *
PamMP (%)	33.2	18.0	46.0	
HSA (°)	164.2	142.0	175.0	0.325
PamHSA (°)	168.5	154.0	175.0	
NSA (°)	150.9	140.0	161.0	0.117
PamNSA (°)	153.1	142.3	163.1	
LatFHG (mm)	4.3	2.2	6.3	<0.001 *
MedFHG (mm)	2.0	0.3	3.6	
ProxFemCapG (mm)	10.3	7.8	13.4	<0.001 *
FemNeckIsmG (mm)	5.7	2.7	9.2	
LatAcetG (mm)	7.3	4.2	11.9	0.107
LatFHG + MedFHG (mm)	6.3	1.1	9.9	

* Indicates a statistically significant *p* value after applying the Bonferroni correction comparing the pamidronate line measurement to the standard bone edge measurement. For example, MP vs. PamMP is significantly different at *p* < 0.001. FemNeckIsmG, femoral neck isthmus growth; HSA, head–shaft angle based on the usual bone landmarks; LatAcetG, lateral acetabular capital growth; LatFHG, lateral femoral head growth; MedFHG, medial femoral head growth; MP, hip migration percentage based on the usual bone landmarks; NSA, neck–shaft angle based on the usual bone landmarks; PamHSA, head–shaft angle based on pamidronate bands; PamMP, hip migration percentage based on pamidronate bands; PamNSA, neck–shaft angle based on pamidronate bands; ProxFemCapG, proximal femoral capital growth.

## Data Availability

Data are available from the corresponding author upon reasonable request.

## Abbreviations

The following abbreviations are used in this manuscript:

CP	Cerebral palsy
FemNeckIsmG	Femoral neck isthmus growth
GMFCS	Gross Motor Function Classification System
HSA	Head–shaft angle
LatAcetG	Lateral acetabular growth
LatFHG	Lateral femoral head growth
MedFHG	Medial femoral head growth
MP	Migration percentage
NSA	Neck–shaft angle
PamHSA	Head–shaft angle based on pamidronate bands
PamMP	Migration percentage based on pamidronate bands
PamNSA	Neck–shaft angle based on pamidronate bands
ProxFemCapG	Proximal femoral capital growth
